# Special Issue “Synthetic Aperture Radar (SAR) Techniques and Applications”

**DOI:** 10.3390/s20071851

**Published:** 2020-03-27

**Authors:** Fabio Bovenga

**Affiliations:** Institute for Electromagnetic Sensing of the Environment (IREA), Italian National Research Council, Via Amendola, 122/d, 70126 Bari, Italy; bovenga.f@irea.cnr.it

**Keywords:** SAR imaging, multi-angle/wide angle SAR, inverse SAR, ground-based SAR, ionospheric effects, SAR interferometry, SAR image analysis, SAR image change detection, SAR sea applications

## Abstract

This editorial of the special issue titled “Synthetic Aperture Radar (SAR) Techniques and Applications”, reviews the nineteen papers selected for publication. The proposed studies investigate different aspects of SAR processing including signal modelling, simulation, image analysis, as well as some examples of applications. The papers are grouped according to homogeneous subjects, then objectives and methods are summarised, and the more relevant results are commented.

## 1. Introduction

Synthetic Aperture RADAR (SAR) became a well-established and powerful remote sensing technology used worldwide for several applications thanks to the possibility of sensing the Earth’s surface at night and day, and in any weather condition. Recent advances have dramatically increased the SAR monitoring potential by improving spatial resolution, revisit time, swath width and polarimetric capability. Moreover, the present and forthcoming spaceborne missions, allow SAR imaging at different bands and acquisition modes (e.g., spotlight, wide swath, bistatic, multi-static and geosynchronous). All these advances stimulated the investigation of new processing algorithms, products, and applications able to fully exploit the new sensor capabilities (e.g., wide spectral band, multi-angle view, short revisit time), as well as the large and continuously updated SAR data archives. The same holds for SAR imaging from ground-based platforms, airplanes and Unmanned Aerial Vehicles (UAVs).

This editorial paper reviews the content of the special issue dedicated to SAR techniques and applications, by presenting advances on SAR signal modelling, SAR simulation, SAR processing, SAR image analysis and SAR-based applications.

## 2. Contributions

The special issue has collected nineteen papers investigating different aspects of SAR processing, SAR image analysis and SAR applications. The contributions cover topics related to multi-angle/wide angle SAR imaging, Doppler parameter estimation, data-driven focusing, Inverse SAR (ISAR) applied to pulsar signal modelling and detection, Ground-Based SAR (GBSAR), near-field Interferometric ISAR, interaction between SAR signal and infosphere, SAR interferometry for ground displacement monitoring, feature extraction, change detection and SAR-based sea applications. In the following, the papers are grouped according to homogeneous subjects, and are reviewed by summarizing objectives, methods and main contributions. Comments are also provided on those research aspects that are more relevant with respect to the state-of-the-art research on SAR processing and applications.

### 2.1. SAR Imaging

The works in [1,2,3,4,5,6] concern several aspects of the SAR imaging. In [1], the authors propose a multi-angle SAR imaging system suited for an ultrahigh speed platform and based on multi-beamforming. By acquiring images at different angles during the same flight, the system allows better characterization of the target on the ground, as well as a simplified motion error compensation. A procedure is proposed aimed at both improving the range migration algorithm and imaging data from different view angles in a unified coordinate system. This provides images with the same resolution, not deformed and scaled, that can be fused quickly and accurately.

The work in [2] also deals with the optimal processing of SAR data acquired with wide aperture, as for circular SAR systems, and in particular tackles the problem of aspect-dependent backscattering. The authors propose an approach based on the least squares of compressed sensing residuals, which is used in video imaging and does not require the isotropic scattering assumption adopted by other methods. This procedure is able to reconstruct time sequences of sparse signals changing slowly with time, and thus it is well suited to processing images derived from SAR sub-apertures, which are highly overlapped. The proposed approach was tested on real data, providing a more accurate estimation of aspect dependent scattering than other methods based on compressed sensing.

The work in [3] concerns the processing of multi-pass squinted SAR data. The proposed algorithm combines images acquired with the same azimuth squint angle on each pass for performing 3D imaging (as in SAR tomography), and data acquired with different azimuth squint angles for refining the suppression of azimuth sidelobes. A performance analysis is carried out by using both simulated point targets and real data acquired by TerraSAR-x satellite mission. The algorithm is able to improve the azimuth suppression while preserving the mail lobe resolution.

The work in [4] considers the problem of Doppler parameter estimation and compensation for SAR data acquired by airborne systems with very high squint geometries. The authors propose an algorithm based on extended multiple aperture mapdrift, which is able to estimate the Doppler phase spatial variation of the third-order. This high order is required for focusing data acquired at high squint angle and at high resolution along very variable aircraft trajectories. In this case, indeed, the inertial navigation system is not able to provide positioning, velocity and angle information accurate enough for a reliable SAR focusing. The method was used for processing both simulated data and real airborne data, and provided accurate targets focusing, thus demonstrating the reliability of high order Doppler parameter compensation.

The imaging of airborne wide-area surveillance (WAS) radar is considered in [5]. For this kind of radar system, Doppler beam sharpening (BDS) imaging is adopted, which, however, suffers from low cross range resolution due to the short dwell time. The authors propose a knowledge-aided DBS processing able to increase the cross-range resolution by a factor of two. The algorithm exploits the strong spatial coherence between adjacent pulses for increasing the number of pulses processed in each coherent interval, thus enhancing the DBS final resolution. A performance analysis was carried out by first using simulated point targets, and then real WAS data. Results demonstrated that the proposed algorithm outperforms other methods adopted in DBS imaging.

The work in [6] proposes an innovative SAR data focusing algorithm, which does not need the knowledge of neither nominal SAR system parameters, nor sensor attitude and trajectory information. The basic idea consists in estimating directly from the data the range and azimuth reference functions needed for SAR focusing, by exploiting, through Singular Value Decomposition, the inherent redundancy present in the SAR raw data. To ensure reliable parameter estimation, strong point scatterers are needed within the imaged scene. This blind focusing could be very useful, for instance, for SAR systems onboard simple aerial unmanned vehicles to be used in real-time and low-cost applications. The algorithm performances were first assessed through simulations, and then SAR data from the ERS mission were processed by using both the proposed algorithm and the standard Range Doppler focusing approach. Results showed a reasonably good quality of the focused image both in amplitude and phase.

The list of papers devoted to SAR imaging concludes with the interesting study in [7], which uses the principles of inverse SAR for modelling signals coming from a pulsar, reflected by space objects (e.g., asteroids), and then detected by a radio telescope on the Earth. Thanks to the coherence of pulsar emissions, pulsar signals can be modelled as monochromatic Gaussian pulses distributed in a time-frequency signal grid. Accordingly, the detected pulsar signals reflected by a moving space object can be considered as a delayed copy of such pulses and modelled within a passive inverse SAR scenario. A range compression approach for this specific ISAR imaging was introduced theoretically and demonstrated through numerical simulations. The Crab Nebula pulsar was considered as emission source. This study can be used for performing space object navigation, localization and imaging based on pulsar emission.

### 2.2. Ground-Based SAR

The special issue includes also three interesting papers [8,9,10] dedicated to ground-based SAR (GBSAR) systems.

The first one [8] is a communication describing the Imaging Multiple-Input Multiple-Output (MIMO) ground-based interferometric radar developed in order to overcome the main limitations of traditional GBSAR systems, which are based on the mechanical movement of the antenna. The proposed system reduces data acquisition time, thus both limiting the atmospheric artefacts and extending the application to vibration measurements. Moreover, the use of independent modules and integrated technologies allows reducing production costs and improving both the transportability and the deployment of the system. The authors introduced the concept and the design of the system, and presented first results derived by using the developed prototype.

The work in [9] concerns a GBSAR system consisting of an antenna on a rotating boom that performs a 360-degree scanning of the surrounding scene, namely, an ArcSAR system. In order to improve the quality of the ArcSAR imaging, the authors propose a refinement of the image focusing by using a digital elevation model (DEM) derived through interferometric processing. The DEM of the scene is first generated by processing ArcSAR interferometric images, and then projected on ground range. Finally, this interferometric DEM is used for enhancing the ArcSAR imaging of the targets on the scene. The authors described the procedure, provided an accuracy analysis and presented results obtained by simulating ArcSAR images according to existing radar amplitude data and an external DEM.

Finally, the work in [10] proposes improving the interferometric near-field 3D imaging by using a multichannel joint sparse reconstruction. The basic idea consists in deriving multichannel signals by dividing the two observed full apertures into sub-apertures. Then, by exploiting the sparsity of the target echo in each channel, the imaging problem is set up as a multichannel joint sparse reconstruction, and the 3D target image of each sub-aperture target is obtained through the improved orthogonal matching pursuit method. Finally, the high-resolution 3D image is derived by synthesizing the 3D images from each sub-aperture. The proposed algorithm improves the imaging accuracy of both strong scattering centres and anisotropic targets. The procedure was tested by using both electromagnetic simulations and real data acquired in an anechoic chamber by using a prototype system.

### 2.3. Ionosphere

This special issue includes also two works investigating the effects of the ionosphere onto SAR imaging from spaceborne platforms.

In [11], the ionospheric scintillation is considered, being the main limiting factor of spaceborne P-band SAR imaging. The sliding spotlight mode, while increasing the azimuth resolution, is particularly affected by scintillation artefacts due to the long integration time. The theoretical analysis of scintillation effects on the P-band spotlight SAR images was performed by introducing a novel scintillation simulator based on the reverse back-projection algorithm. SAR raw data for the sliding spotlight mode were simulated for both pointy and extended targets, under different scintillation conditions. Simulations allowed investigating the image degradation in azimuth induced by scintillation, and deriving threshold values of scintillation strength and spectral index, which guarantee acceptable P-band spotlight imaging.

One of the most relevant characteristics of the ionosphere is the total electron content (TEC), which is investigated in [12] by exploiting spaceborne polarimetric SAR (PolSAR) data. First, the authors assessed the precision of SAR-based TEC retrieval by comparing measurements derived from L-band PolSAR data acquired by the ALOS satellite, and from very accurate incoherent scatter radars. Then, the TEC of the topside profile was estimated by subtracting from the TEC derived by PolSAR, the TEC of the bottom side profile measured by an ionosonde observing the same space of the satellite. This procedure allows refining the topside TEC estimations and thus improves the modelling of the electron density topside profile.

### 2.4. Ground Displacement Monitoring

The following seven papers are specifically dedicated to applications. The first two [13,14] use SAR interferometry (InSAR) for investigating displacements related to land subsidence and highway deformation. Multi-temporal InSAR is a well-established technique currently applied to displacement monitoring thanks to the availability of reliable processing tools developed during the last two decades, as well as data archives continuously updated by operative satellite missions. Nowadays, there is an increasing need for advanced, application-oriented procedures for analysing the InSAR-based displacement records.

In [13], high-resolution RADARSAT-2 SAR data were processed through the Small BAseline Subset (SBAS) algorithm for deriving 3-year displacement time series over Wuhan city, which suffers from subsidence problems related to urban construction. First, the InSAR results were compared to measurements from levelling benchmarks for a quality check. Then, the mean displacement maps and time series were analysed for studying the subsidence characteristics in space and time. Thanks to the availability of data covering the whole urban area with unprecedented spatial and temporal density, it was possible to provide a reliable assessment of subsidence causes.

The work in [14] proposes an interesting method for improving analysis and interpretation of InSAR displacement products. It consists of modelling the displacement time series of man-made structures by using rheological parameters, namely, viscosity and elasticity, which are engineering properties of the soil allowing to define quantitatively how materials deform in response to forces. The SBAS algorithm was used for processing high-resolution TerraSAR-X data, and then for monitoring a highway segment built on soft clay and affected by deformation. Results were validated by using independent levelling measurements, demonstrating that the proposed modelling method allows improving the estimation of the nonlinear component of the deformation.

### 2.5. Target Discrimination and Change Detection

Thanks to the information content available under all-weather conditions and also during night-time, SAR is widely used for target detection, classification and change detection. The following two papers [15,16] concern this kind of applications.

The work in [15] proposes the use of aspect entropy for quantifying the degree of anisotropy in SAR backscattering, and thus improving the detection of anisotropic targets and their classification. The proposed method applies to SAR systems acquiring under different look angles, as circular SAR. First, the use of aspect entropy as a reliably index of anisotropy was verified in simulations. Afterwards, the authors described the algorithm, which consists of computing the aspect entropy at pixel level for distinguishing between isotropic and anisotropic scattering mechanisms, and then at target level for target classification. Moreover, a denoising method for the radar cross section curve was proposed. Finally, the procedure was validated by using X-band data acquired by a circular SAR.

The work in [16] experiments with the use of two-colour multiview (2CMV) advanced geospatial information products for detecting changes between SAR images acquired at different times. The proposed procedure consists of a pre-processing step for denoising SAR amplitude, followed by the change detection step performed by running algorithms of unsupervised feature learning (K-SVD) and clustering (k-means). Moreover, an optical flow algorithm is used for distinguishing changes related to actual target motions (correct detections) from those related to errors in image co-registration (false positives). The procedure was first introduced, and then tested on datasets coming from both an airborne high-resolution X-band SAR, and the spaceborne medium resolution C-band ERS-2 mission. Results were also compared with other methods, showing improvements in presence of co-registration and perspective errors.

### 2.6. Sea Applications

The last three papers [17,18,19] of the special issue present SAR imaging algorithms devoted to two sea applications, namely, ship detection and classification, and oceanic eddy detection and analysis.

The work in [17] presents a new method for refocusing moving ships in SAR images. Ship detection is widely required for both civilian and military surveillance; however, SAR images derived by standard focusing suffer from strong blurring in presence of moving ships. Therefore, a further processing step is needed, which performs reliable motion compensation and refocusing. The paper proposes an algorithm based on an inverse SAR technique able to refocus only the portions of SLC matrix containing moving ships, instead of the whole raw image. Motion phase compensation is performed through an iterative procedure based on a fast minimum entropy method. The procedure was presented and validated by using both airborne data and spaceborne images acquired by TerraSAR-X and Gaofeng-3 missions. Results showed improvements with respect to other refocusing methods.

The work in [18] also deals with ship detection. It proposes a method that performs ship classification by processing SAR images through convolutional deep neural networks (CNN). In this application contest, the SAR datasets available for training the network are often limited, whereas CNNs require thousands of examples to avoid overfitting. In order to overcome this problem, the proposed algorithm starts with an augmentation method for enlarging the training dataset. Then, transfer learning is used for improving the classification accuracy. The procedure was tested by processing TerraSAR-X high-resolution images. Results were compared with outcomes coming from other classification methods, showing improved performances.

Spaceborne SAR observations are very promising for identifying and studying the mechanism governing oceanic eddies. The last paper [19] of this special issue concerns SAR imaging of oceanic eddies generated by shear-waves. The authors developed a method for simulating the current field of an ocean eddy according to the Burgers–Rott vortex model. These simulated ocean eddies were then used to generate SAR images through a simulation tool. The developed procedure is able to perform simulations under different geometric and radiometric SAR configurations, and different wind conditions (speed and direction). Results were validated by using real SAR data provided by ERS-2 and ENVISAT satellite missions. This kind of tool results very useful to understand how radar and wind characteristics impact on the eddy features in SAR images, and thus to support their interpretation and study.

## 3. Conclusions

In this editorial paper, we reviewed the content of the special issue dedicated to SAR techniques and applications. All the selected nineteen papers proposed interesting advances on different aspects of the SAR processing concerning signal modelling, imaging simulation, image analysis and some applicative examples. In particular, issues were addressed related to multi-angle and wide-angle acquisition modes, which, in the last years, have been becoming more and more common. Several SAR systems were considered, including spaceborne and aerial platforms, light unmanned vehicles, as well as ground-based radars. Specific aspects of SAR signal propagation (e.g., in ionosphere) and back-scattering (e.g., anisotropic backscattering, ocean eddies) were also investigated though modelling and simulation. Moreover, an interesting study proposed modelling pulsar signals by using inverse SAR imaging principles. Applicative examples were also presented based on advanced techniques for image analysis and signal modelling, including, among others, convolutional deep neural networks, unsupervised feature learning and clustering, and deformation modelling through rheological parameters.

In conclusion, the proposed studies represent valid examples of the fertile research ongoing in the field of SAR processing and applications, and demonstrate as SAR imaging still presents large margins for investigations.
